# Laparoscopic intraoperative pancreatoscopy for main duct intraductal papillary mucinous neoplasms assessment

**DOI:** 10.1016/j.vgie.2022.09.002

**Published:** 2022-11-19

**Authors:** Abdellah Hedjoudje, Safi Dokmak, Jérôme Cros, Alain Sauvanet, Frédéric Prat

**Affiliations:** 1 Service d’Endoscopie Digestive, DMU DIGEST, Beaujon Hospital, Assistance Publique Hopitaux de Paris (APHP), Clichy, France; 2 Service de Pancréatologie, DMU DIGEST, Beaujon Hospital, Assistance Publique Hopitaux de Paris (APHP), Clichy, France; 3 Service de Chirurgie Hépato-Biliaire, DMU DIGEST, Beaujon Hospital, Assistance Publique Hopitaux de Paris (APHP), Clichy, France; 4 Service d’Anatomopathologie, Beaujon Hospital, Assistance Publique Hopitaux de Paris (APHP), Clichy, France; 5 Service de Chirurgie Hépato-Biliaire, DMU DIGEST, Beaujon Hospital, Assistance Publique Hopitaux de Paris (APHP), Clichy, France; 6 Service d’Endoscopie Digestive, DMU DIGEST, Beaujon Hospital, Assistance Publique Hopitaux de Paris (APHP), Clichy, France

**Keywords:** IOP, intraoperative pancreatoscopy, IPMN, intraductal papillary mucinous neoplasm of the pancreas, MD-IPMN, main pancreatic duct intraductal papillary mucinous neoplasms, MPD, main pancreatic duct

## Abstract

Video 1 Intraoperative pancreatoscopy during laparoscopic pancreatic resection for main pancreatic duct intraductal papillary mucinous neoplasms.

Intraoperative pancreatoscopy during laparoscopic pancreatic resection for main pancreatic duct intraductal papillary mucinous neoplasms.

Intraductal papillary mucinous neoplasms of the pancreas (IPMNs) are increasingly diagnosed tumors that are characterized by endoluminal papillary projections of mucin-producing ductal epithelium, leading to a dilatation of the ducts it develops within. IPMNs carry a high risk of malignancy when the main pancreatic duct is involved (MD-IPMNs). When feasible, surgical resection of the pancreatic segment involved is the therapeutic option of choice. Complete resection of IPMN lesions is essential and requires precise diagnosis of the extent of the disease. However, it can be difficult to evaluate the involvement of the pancreatic duct especially to differentiate “passive” dilatation upstream of IPMN foci from dilatation on the site of discrete MD-IPMN. Surgical studies found that mild to moderate dysplasia can be found on the resection margin in 40% of patients.^1^

Peroral pancreatoscopy (performed endoscopically during an ERCP) has been proposed to improve the accuracy of preoperative workup.^2^^,^^3^ However, single-operator peroral pancreatoscopy is technically challenging and not without risks, especially when the main pancreatic duct (MPD) is not dilated or moderately dilated.^3^ Intraoperative pancreatoscopy (IOP) can be used to evaluate the main pancreatic duct intraoperatively to help determine the extent of pancreatic duct involvement and tailor resection to the affected segment, thus sparing the unaffected pancreas.^4^^,^^5^

We herein present the case of a 50-year-old man with a history of spondyloarthritis and tobacco addiction. He was found to bear a diffusely dilatated main pancreatic duct up to 15 mm over the body and tail portions. Endoscopic ultrasound and magnetic resonance imaging showed an 18-mm mural nodule in the head of the pancreas and a diffusely dilatated pancreatic duct without obvious lesions in the body and tail of the pancreas. A 3-dimensional laparoscopic pancreatoduodenectomy was planned, and a preoperative pancreatoscopy was discussed during the multidisciplinary team meeting. Intraoperative pancreatoscopy was the preferred option to rule out high-risk lesions beyond the intramural nodule identified on imaging. After transsection of the pancreas under laparoscopy, the Spyglass Discover cholangiopancreatoscope (Boston Scientific, Voisins-le-Bretonneux, France) (Table 1, Fig. 1) was inserted through a 5-mm trocar and exploration of the main pancreatic duct was performed. The surgeon manipulated the tip of the scope with forceps to facilitate introduction into the main pancreatic duct, and then manipulation was achieved by the endoscopist. Back and forth inspection of the MPD over its full length was achieved under saline irrigation. Particular attention was given to putative skip lesions as well as to the portion of the duct closest to transsection. In the present case, exploration of the MPD and the distal pancreas revealed a dilated tail of the pancreatic duct, as well as side-branches and accessory ducts that were otherwise normal. After this examination was completed, the same procedure was done on the opposite, tumor-involved side, with determination of the distance between the last visible papillary projections and the transsection line. The pancreatoscope was passed proximally in the direction of the ampulla of Vater from the point of parenchymal transection and slowly removed, assessing the remnant duct (in the head of the pancreas) in a similar fashion. This part of the exploration revealed a dilated pancreatic duct with papillary, fish egg–like protrusions, adherent mucin, and nodularity. Pathology examination reported high-grade, noninvasive, intestinal-type IPMN extending over 35 mm with a 15-mm safe R0 resection margin and mild chronic pancreatic change in the adjacent pancreas (Fig. 2A and B). The patient experienced a grade A biliary leak and a mild postoperative reactivation of spondylarthritis leading to a delayed discharge at 21 days (Video 1, available online at www.giejournal.org).

**Table 1:** SpyGlass Discover characteristics

Direction of view	0 degrees (forward viewing)
Field of view	120 degrees
Distal tip width	10.5F (3.5 mm)
Maximum insertion width	10.8F (3.6 mm)
Working length	65 cm
Minimum accessory channel width	3.6F (1.2 mm)
Minimum angulation range	30 degrees with accessory device in working channel

**Figure 1:**
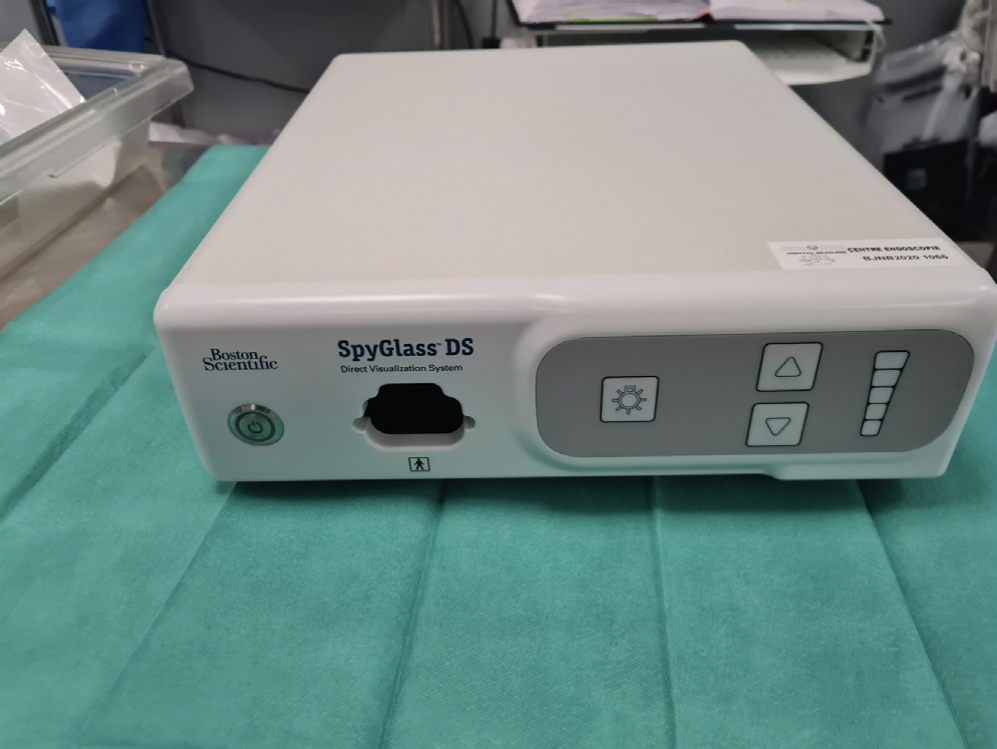
The SpyGlass Discover system comprises the Discover scope, a disposable, single-use, sterile device, and the SpyGlass Discover Controller.

**Figure 2:**
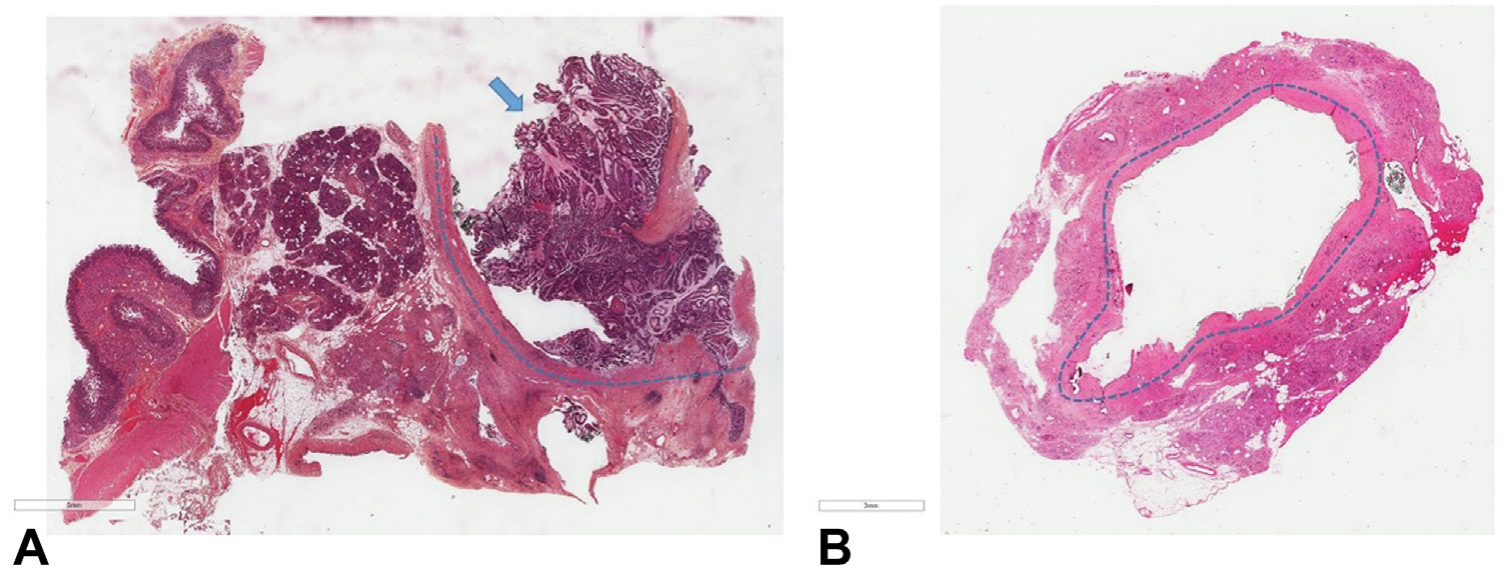
**A**, H&E slide. Large nodule (*blue arrow*) corresponding to the IPMN within the main duct (duct limit highlighted by a *blue dotted line*) (H&E, orig. mag. ×10). **B**, H&E slide. Isthmic left section on the surgical specimen showing a dilatated main duct (circled by a *blue dotted line*) without IPMN lesion (H&E, orig. mag. ×10). IPMN, Intraductal papillary mucinous neoplasm of the pancreas.

We hereby found that intraoperative pancreatoscopy seems feasible and useful for determining the therapeutic approach. In a previous study conducted by Hara et al,^6^ authors found that identification of fish-egg-like protrusions with vascular images, villous protrusions, or vegetative protrusions allows discrimination of malignant from benign main duct IPMNs with an accuracy of 88%. Tyberg et al^3^ reported the first use of digital single-operator cholangiopancreatoscopy for presurgical mapping of pancreatobiliary malignancy and found that 62% of their patients undergoing surgery for IPMN had a change in their surgical plan based on preoperative pancreatoscopy. Of these, half required more extensive surgery and half required less extensive surgery. In another previous study using a flexible endoscope conducted by Navez et al,^7^ 21 patients with a dilated main pancreatic duct had intraoperative pancreatoscopy using an ultrathin flexible endoscope and biopsy forceps, and specimens of all suspicious lesions underwent frozen section examination. A complete intraoperative pancreatoscopy with intraductal biopsies was easily and safely performed in 21 patients, revealing 8 occult IPMN lesions. In 5 cases (23.8%), initially planned surgical resection was modified secondary to IOP: 3 for carcinoma in situ and 2 for invasive carcinoma. In another study conducted by Pucci et al,^8^ 23 cases had intraoperative pancreatoscopy with a 7.5F (3-mm diameter) flexible choledochoscope (Karl Storz, Tuttlingen, Germany). Eighteen (78%) of these operations were performed for presumed MD-IPMN. In 5 cases (22%), the surgical resection was extended secondary to the intraoperative pancreatoscopy findings. Current guidelines recommend the use of frozen section analysis of the pancreatic resection margin for all partial pancreatectomies and parenchyma-sparing pancreatectomy in patients with IPMN to drive the extent of resection.^9^ However, the attitude to adopt based on pathology finding remains controversial. While most surgeons agree that further resection is warranted in cases of high-grade dysplasia or cancer, the presence of lower grades of dysplasia is debated. Half of surgeons believe that an additional resection is not required.^10^ In case of denuded epithelium hindering a proper pathological assessment, while 34% felt that a further resection is typically not necessary, 57% would proceed with one. Also, frozen sections are unable to detect the presence of skip lesions responsible for neoplastic recurrences despite negative resection margins.^11^ Thus, the additional information provided by the IOP combined with intraoperative frozen section can help to choose the optimal operative strategy and decrease recurrence rate.

In conclusion, our case shows the benefit of digital pancreatoscopy in MD-IPMN when the pancreatic duct is diffusely dilated without focal findings on cross-sectional imaging or EUS. Prospective studies will be needed to confirm our finding of a benefit for intraoperative pancreatoscopy.

## Disclosure

*The authors disclosed no financial relationships.*

## Supplementary data

Video 1 Intraoperative pancreatoscopy during laparoscopic pancreatic resection for main pancreatic duct intraductal papillary mucinous neoplasms.

## References

[1] Falconi M., Salvia R., Bassi C. (2001). Clinicopathological features and treatment of intraductal papillary mucinous tumour of the pancreas. Br J Surg.

[2] Trindade A.J., Benias P.C., Kurupathi P. (2018). Digital pancreatoscopy in the evaluation of main duct intraductal papillary mucinous neoplasm: a multicenter study. Endoscopy.

[3] Tyberg A., Raijman I., Siddiqui A. (2019). Digital pancreaticocholangioscopy for mapping of pancreaticobiliary neoplasia: can we alter the surgical resection margin?. J Clin Gastroenterol.

[4] Del Chiaro M., Verbeke C., Salvia R. (2013). European experts consensus statement on cystic tumours of the pancreas. Dig Liver Dis.

[5] Shah R.J. (2015). Innovations in intraductal endoscopy: cholangioscopy and pancreatoscopy. Gastrointest Endosc Clin N Am.

[6] Hara T., Yamaguchi T., Ishihara T. (2002). Diagnosis and patient management of intraductal papillary-mucinous tumor of the pancreas by using peroral pancreatoscopy and intraductal ultrasonography. Gastroenterology.

[7] Navez J., Hubert C., Gigot J.-F. (2015). Impact of intraoperative pancreatoscopy with intraductal biopsies on surgical management of intraductal papillary mucinous neoplasm of the pancreas. J Am Coll Surg.

[8] Pucci M.J., Johnson C.M., Punja V.P. (2014). Intraoperative pancreatoscopy: a valuable tool for pancreatic surgeons?. J Gastrointest Surg.

[9] European Study Group on Cystic Tumours of the Pancreas (2018). European evidence-based guidelines on pancreatic cystic neoplasms. Gut.

[10] Marchegiani G., Salvia R., Verona E.B.M., 2020 on IPMN (2021). Guidelines on pancreatic cystic neoplasms: major inconsistencies with available evidence and clinical practice - results from an international survey. Gastroenterology.

[11] Eguchi H., Ishikawa O., Ohigashi H. (2006). Role of intraoperative cytology combined with histology in detecting continuous and skip type intraductal cancer existence for intraductal papillary mucinous carcinoma of the pancreas. Cancer.

